# Acute metformin treatment increased social sniffing in male but not female BTBR mice

**DOI:** 10.3389/adar.2026.16883

**Published:** 2026-10-12

**Authors:** Susan M. Greene, Gloria-Andrea Alcala, Blanka Bordas, Vy Vu, Yatzil Sanchez, Angela Ontanon, Georgianna G. Gould

**Affiliations:** 1 Center for Biomedical Neuroscience and School of Medicine, The University of Texas Health Science Center at San Antonio, San Antonio, TX, United States; 2 University of the Incarnate Word, San Antonio, TX, United States; 3 Burrell College of Osteopathic Medicine, Las Cruces, NM, United States; 4 Virginia Tech, Blacksburg, VA, United States

**Keywords:** metformin, mouse model of autism, sex differences, social behavior, steroids

## Abstract

Autism spectrum disorders (ASDs) are characterized by social deficits and restrictive-repetitive behaviors. Metformin has recently been investigated for its potential behavioral effects in ASD models. This study examined the acute effects of metformin on social interaction preferences and marble burying in male and female BTBR T + Itpr3tf/J (BTBR) mice, with C57BL/6J and 129S1/SvImJ mice included as strain controls. The mice received a single intraperitoneal injection of metformin (0 mg, 75 mg, or 150 mg/kg), and the effects were compared using three-chamber social preference and marble burying tests. Acute metformin increased not only social interaction preferences, as determined by differences in time spent in chambers, but also by differences in social sniffing time in male BTBR mice at 150 mg/kg. Surprisingly, it did not alter sociability preferences in BTBR females or in C57BL/6J or 129S1/SvImJ mice. In C57BL/6J females, metformin reduced chamber entries and marble burying, whereas males were unaffected. Progesterone and corticosterone levels were measured in BTBR and C57BL/6J serum collected after behavioral testing, and their levels were unaffected by metformin treatment. These findings indicate that metformin acutely produces modest, beneficial behavioral effects in male BTBR mice, which otherwise exhibit social interaction deficits that are translationally relevant to ASD.

## Graphical Abstract

**Figure:**
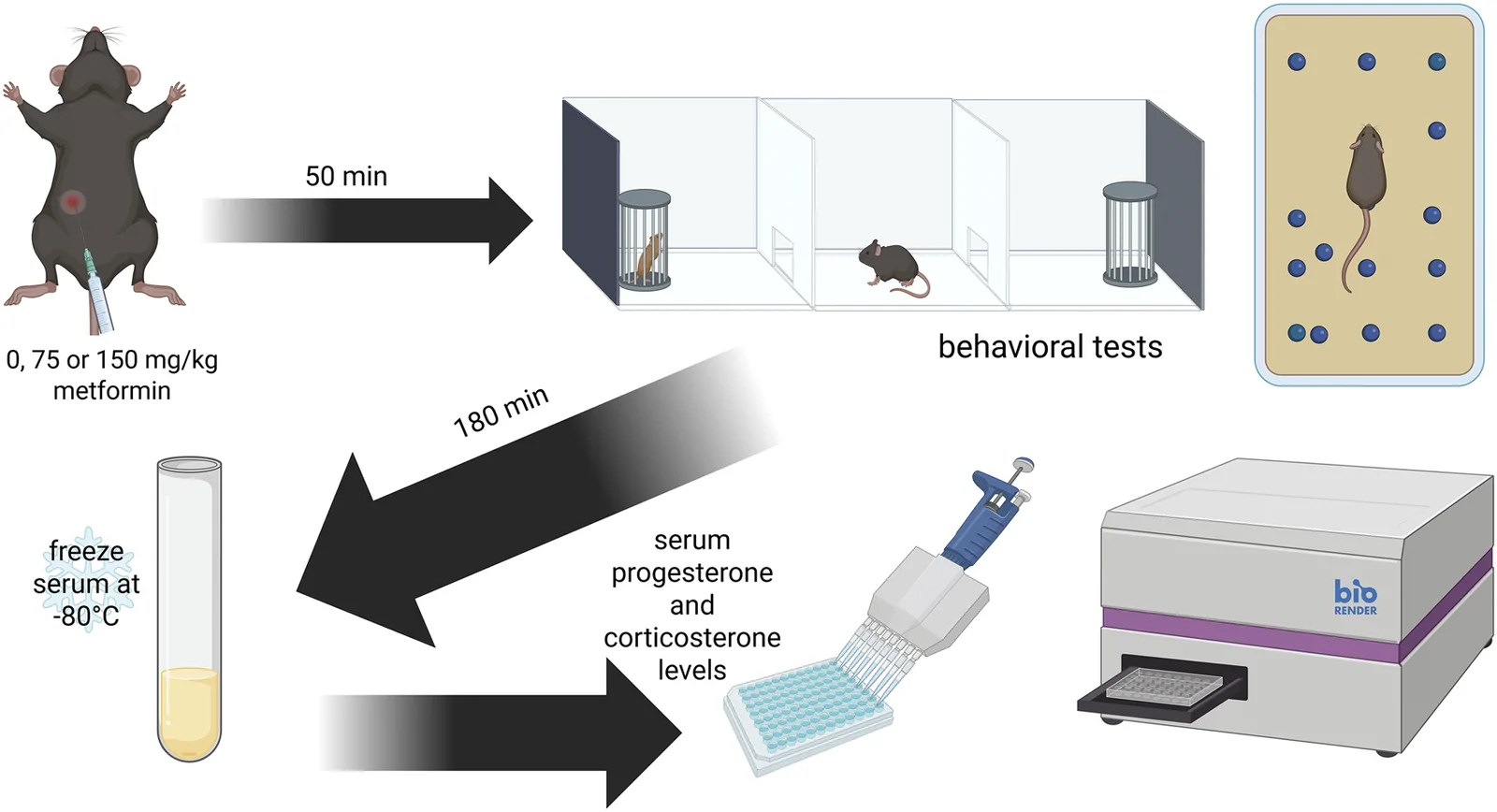
Acute metformin treatment study timeline. Created in BioRender. Gould, G. (2026) https://BioRender.com/gifgqv8.

## Highlights

- Metformin enhanced social interaction preference in male but not female BTBR mice
- In C57BL/6J females, metformin reduced test arena exploration and marble burying
- In 129S1/SvIMJ mice, metformin had no impact on social preference or marble burying
- Progesterone and corticosterone levels were unchanged by acute administration of 75 mg or 150 mg/kg metformin

## Introduction

Autism spectrum disorders (ASDs) are lifelong neurodevelopmental conditions commonly diagnosed in childhood when afflicted with both restricted and repetitive traits, along with social deficits [1, 2]. In addition to the obvious behavioral symptoms of autism, physiological changes have also been noted, such as elevated levels of steroid hormones such as cortisol, testosterone, and progesterone [3–6]. These physiological changes are often overlooked when managing behavioral symptoms despite their potential utility as biomarkers.

Due to the complex nature of autism, which has both environmental and genetic factors, finding a comprehensively effective treatment can be difficult [7–11]. Typically, this condition is treated using atypical antipsychotic drugs to target both core symptoms, with common prescriptions including aripiprazole and risperidone [1, 12, 13]. However, one unfortunate side effect of antipsychotic treatments is persistent weight gain. A variety of clinical trials and testing with mouse models have concluded that metformin is an effective treatment for reducing the metabolic changes and weight gain often arising from taking antipsychotic drugs [1, 12–18].

Recent studies have started investigating the effects of metformin as a treatment for symptoms of ASD, showcasing metformin’s effectiveness in alleviating symptoms without the need for the antipsychotic drugs often paired with it [1]. When metformin enters the body, it is transported by the gut organic cation transporter 1 (OCT1) and organic cation transporter 3 (OCT3) to the liver, placenta, and brain [10, 19]. In the liver, metformin enters the mitochondria via OCT1, where it acts on the AMP-activated protein kinase (AMPK) to ramp up ATP production while simultaneously inhibiting complex 1 of the mitochondria, ultimately blocking ATP production and inhibiting gluconeogenesis, resulting in weight loss and reduced blood sugar [19, 20].

While metformin is widely prescribed to treat diabetes and increase insulin sensitivity, it also reduces androgen levels and/or androgen receptor expression [19, 21]. Additionally, metformin inhibits melanocortin receptor 2 (MC2R) to attenuate the response to adrenocorticotropic hormone (ACTH), thus reducing adrenal synthesis and release of cortisol and progesterone [22]. Currently, metformin is prescribed to lower androgens to promote fertility in women with polycystic ovary syndrome (PCOS) [22, 23]. In addition to reducing weight, blood glucose, and adrenal steroids, metformin treatment was found to reduce anxiety, enhance cognition, lower serum corticosterone, increase brain 5-HT, and enhance gut function [1, 2, 24–28]. A common phenotypical model of ASD, the BTBR mouse, exhibits impaired social interaction preference and restrictive-repetitive behaviors, such as increased grooming and marble burying [2, 26, 28]. When given metformin, male BTBR mice had increased social behavior in the three-chamber test for social interaction, spending more time with stranger mice than those not treated with metformin [2, 28]. Some studies have found that male BTBR mice buried fewer marbles and spent less time grooming after metformin treatment than untreated mice, while other studies have shown that metformin has no impact on restrictive-repetitive behaviors [2, 26, 28]. Thus, metformin can reduce ASD-relevant traits in BTBR males.

Given the improvements in social behaviors and a decrease in restrictive-repetitive traits observed with chronic metformin treatments in BTBR mice in previous studies [2, 28], this study examines the immediate behavioral and hormonal effects of metformin after acute treatment. While the majority of studies have focused on the impact of metformin after chronic treatment, few have addressed its more immediate effects. This study also uses both male and female mice, as previous studies had only tested male mice [2, 26–28]. Three separate strains of mice were selected to measure changes in social behavior and restrictive-repetitive traits after acute treatment with metformin. BTBR mice were used as the ASD model, and C57BL/6J (C57) mice were used as the control strain. After metformin treatment, steroid hormones were measured in both strains. Finally, the 129S1/SvImJ (129S) strain was chosen as an alternate control strain to measure metformin’s effect due to its close genetic relationship and potential historical cross-breeding with both the C57 and BTBR mouse strains [29]. It was hypothesized that BTBR mice would show dose-dependent improvements in social behavior and a decrease in restrictive-repetitive traits after acute metformin treatment, while control strains would be unaffected.

## Materials and methods

### Animals

The subjects were 213 mice; 36 BTBR T + Itpr3tf/J (BTBR, JAX stock #002282) females weighing (mean ± standard deviation (SD)) 28 ± 4 g and 39 males 34 ± 5 g, 36 each C57BL/6J (C57, JAX stock #000664) females weighting 23 ± 4 g and males 32 ± 5g, and for 129S1/SvImJ (129S, JAX stock #002448) 30 females weighing 22 ± 3 g and 36 males weighing 28 ± 4 g. All mice were bred in-house from founders originating from Jackson Laboratories (Bar Harbor, ME, USA). The housing room temperature was between 22 °C and 25 °C with a 14:10 light-dark cycle (300 lux, as measured by the Lux Light Meter Pro App for iOS) on at 0700 h. The cages were JAG75 cages (29 × 18 × 12 cm), which were sterilized, contained wood-chip bedding (Teklad, Harlan, Indianapolis, IN), and were stored on ventilated housing racks (Micro-Vent 140-cage mouse IVC model for JAG75, 60 air cages/h from Allentown, Inc., Allentown, PA, USA). All cages contained 2-5 same-sex mice of the same strain with up to a one-week age difference, *ad libitum* access to chow (Teklad #7912, Harlan, Madison, WI) and water (reverse-osmosis and acidified to a pH of 2.5–3.0 using HCl). Separate same-sex 129S mice not tested in behavioral tests were used as stranger mice for sociability preference tests for BTBR and C57 mice, while same-sex C57 mice were used as strangers for 129S mice. To prevent familiarization with these animals, they were stored on a separate ventilated rack in the same room. All mice were moved to a behavior testing room to acclimate for 1 h in their home cages on a cart. The animals were humanely euthanized via cervical dislocation and decapitation 3 h after acute injection, and serum was isolated from clotted trunk blood by centrifugation at 2,500 × g and kept at −80 °C. All procedures involving live mice complied with the ARRIVE guidelines, in addition to the National Institutes of Health Guide for the Care and Use of Laboratory Animals (Publication #8023). These procedures were also approved by the University of Texas Health Science Center at San Antonio’s Institutional Animal Care and Use Committee.

### Drug management and preparation

Metformin hydrochloride was obtained from Enzo Life Sciences, Inc., Farmingdale, New York, USA (Cat. No.: ALX-270-432-G005, Lot No.: 12211506, stored at 4 °C). The drug was stored between −20 °C and 4 °C depending on the supplier’s recommendations before being made into a stock solution for acute intraperitoneal (i.p.) injections.

For acute treatment, animals were injected intraperitoneally (i.p.) with either 0 mg, 75 mg, or 150 mg/kg of metformin in a saline vehicle. The tails of the animals were marked with a permanent marker to distinguish them after each one was weighed on a digital scale. They were injected in a dimly lit (16 lux red light) behavioral testing room and sat in their home cages for 30 min post-injection before undergoing behavioral tests.

### Social interaction and novelty

Social interaction and novelty preferences were measured in a three-chamber test [30–32]. Age- and weight-matched 129S stranger mice were used with BTBR, and C57 mice, while C57 stranger mice were used with 129S mice. All stranger mice were pre-conditioned under a wire mesh cup for 30 min before testing began. All testing took place between 900 h and 1800 h, with low red lighting (16 lx, as measured by the Lux Light Meter Pro App for iOS), in groups of no more than 6 mice at a time. Subject mice were preconditioned for 10 min in the middle chamber, followed by another 10 min of preconditioning in all three chambers, before the test began. During social interaction testing, a same-sex stranger mouse was placed in one end chamber while an empty cup cage novel object was placed in the opposite end chamber. Social novelty tests were performed directly afterward by introducing a new stranger of the same sex under the empty wire cup while the original stranger remained unmoved. Video recordings were taken of the mice for 10 min during the interaction and novelty tests to measure time spent in the chamber, entries into the chamber, and time spent sniffing either the stranger mouse or novel object. All behaviors were measured by treatment-blind observers using handheld timers. Preference scores for time in the chamber and time spent sniffing were then calculated by subtracting the time spent in the chamber or sniffing the novel object (empty cage) from the time spent in the chamber or sniffing the stranger mouse, or the time spent in the chamber or sniffing the “old” or original stranger mouse encountered in the interaction preference test from the time spent in the chamber or sniffing the new stranger mouse.

### Marble burying

Marble burying was conducted as a measure of restrictive-repetitive traits and performed directly after the social novelty test [32, 33]. Briefly, 40 × 20 cm sterilized rat cages filled to a depth of 8 cm with wood chip bedding were used, with 18 flat blue glass marbles added in 3 rows of 6. Mice were given 30 min to bury the marbles, starting at the time when they entered the cage. Room lighting was the same as in the sociability tests (16 lx, as measured by the Lux Light Meter Pro App for iOS). Marbles were counted as buried if they were two-thirds buried in the wood chip bedding.

### Serum steroid hormones

Serum from a subset of BTBR (N = 4–12) or C57 (N = 6–10) mice, which was stored at −80 °C, was used for hormone measurements. The serum was thawed and diluted 1:30 in assay buffer before use. Progesterone and corticosterone were measured using enzyme-linked immunosorbent assay kits (Cat. No.: 582601 and 501320, respectively, Cayman Chemical Co., Ann Arbor, MI, USA).

### Statistical analysis of data

Social interaction and novelty test data were analyzed for all strains using a two-way analysis of variance (ANOVA, for metformin dose x sex) for each strain to detect differences in time spent in the chamber or time spent sniffing for social interaction (stranger–empty cage) or social novelty (new stranger–original stranger) preferences, time spent in the middle chamber, chamber entries, and marble burying. Tukey’s *post hoc* test, controlling for multiple comparisons, was used for all significant main effects of dose, sex, chamber, or interactions. All main figures show minimum-to-maximum box plots, and the supplementary figures show the individual data points analyzed using a two-way mixed model within-groups ANOVA followed by Šídák’s multiple comparisons test. All statistics and graphs were obtained using Prism (version 10 for Windows; GraphPad, LaJolla, CA, USA).

## Results

### Social interaction preference

#### BTBR mice

For BTBR mice, the two-way ANOVA interaction for time spent in chambers was significant (F (2, 69) = 3.3, p = 0.04). Males on 150 mg/kg metformin showed a greater preference for social interaction than either females on 150 mg/kg metformin (p < 0.01), or male saline controls (p = 0.02) (Figure 1A). Preference for social sniffing during interaction tests was significantly greater in males on 150 mg/kg metformin (F (2, 69) = 3.9, p = 0.02) than in saline-treated males (p = 0.04) and females ((p < 0.01) on the same 150 mg/kg dose of metformin (Figure 1B). No significant effects were detected for the number of chamber entries (Figure 1C) or the time (mean ± SD = 69 ± 52 s) spent in the middle chamber.

**FIGURE 1:**
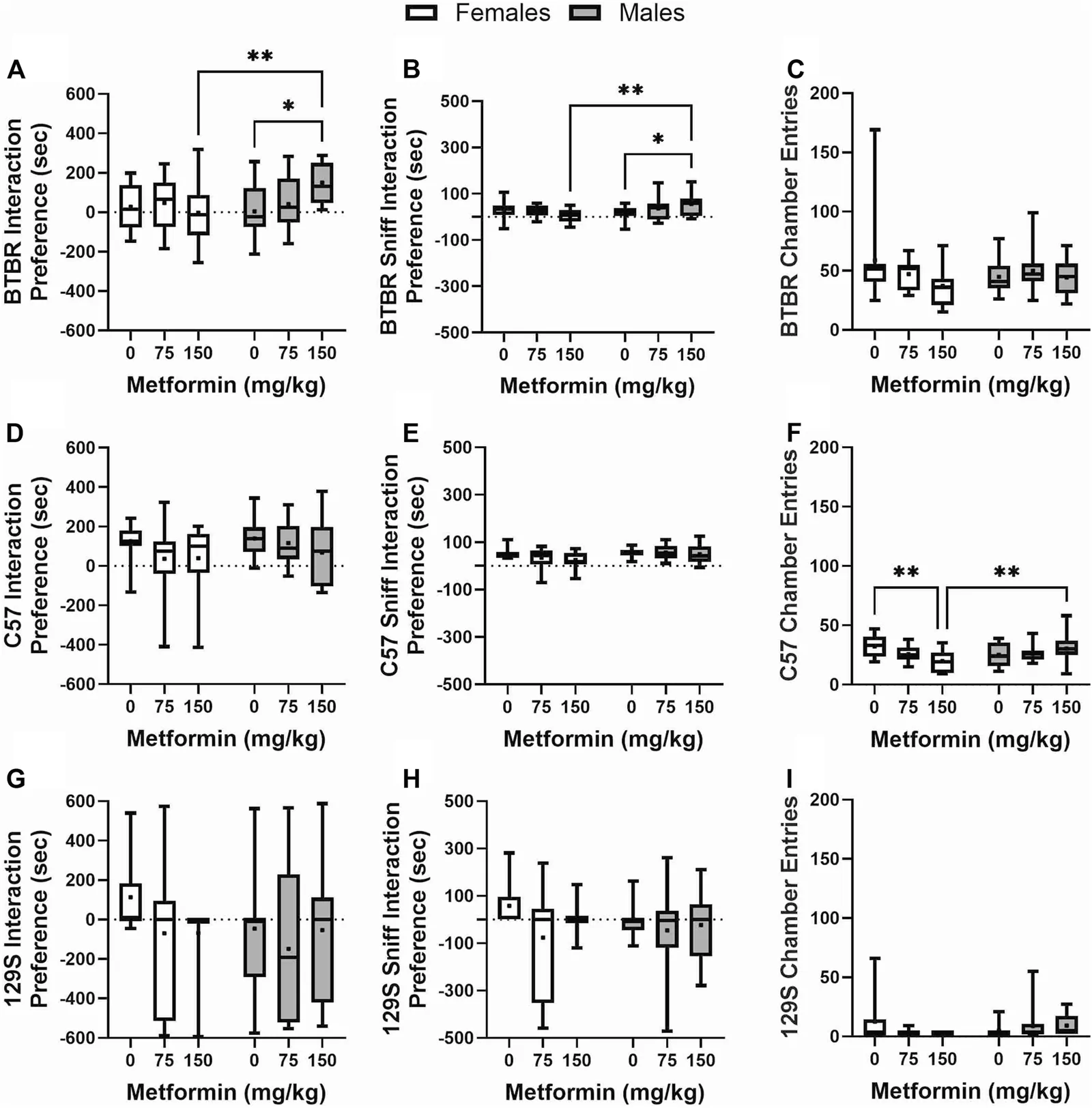
Social interaction preferences, based on differences in time spent in chambers with or sniffing stranger mice versus a novel object (empty cup cage), and chamber entries of BTBR, C57, and 129S mice 50–60 min after acute metformin treatment. The data shown are minimum-to-maximum interquartile range box (females = no fill, males = gray) and whisker plots with dots at the means and lines at the medians for the 0 (control), 75 mg, and 150 mg/kg doses of metformin. BTBR males on 150 mg/kg metformin spent more time near **(A)** and sniffing **(B)** stranger mice than novel objects as compared to controls (*p < 0.05) or females on 150 mg/kg metformin (**p < 0.01), with no metformin effects on females, and **(C)** no changes in chamber entries indicative of altered exploratory locomotion. C57 mice did not respond to metformin treatment in terms of **(D)** time spent in the chamber or **(E)** sniffing time. Female C57 mice on 150 mg/kg metformin made fewer **(F)** chamber entries than either control females or males on 150 mg/kg metformin (**p < 0.01).The behavior of 129S mice in social preference tests was highly variable, and no responses to metformin were detected in terms of **(G)** time spent in the chamber, **(H)** sniffing time,or **(I)** chamber entries. Sample sizes are as follows: BTBR females = 12, males = 13, C57 females and males = 12, 129S females = 10, males = 12.

#### C57 mice

For C57 mice, there were no significant interactions or effects of sex or metformin dose on social interaction preference based on time in chambers (Figure 1D). For time engaged in social sniffing, there was an effect of sex (F (1, 66) = 4.5, p = 0.04), which was not significant in *post hoc* multiple comparisons (Figure 1E). There were no sex- or treatment-related differences in time spent in the middle chamber (mean ± SD = 116 ± 50 s). However, there was a sex × dose interaction observed for C57 chamber entries (F (2, 66) = 5.5, p < 0.01), as 150 mg/kg metformin-treated females exhibited lower locomotor exploration of the three-chamber arena than control females (p < 0.01) and 150 mg/kg-treated males (p < 0.01), as shown in Figure 1F.

#### 129S mice

For 129S mice, there were no interactions or effects of sex or metformin dose for interaction preference based on time in chambers (Figure 1G), sniffing times (Figure 1H), or chamber entries (Figure 1I). There was an effect of sex (F (1, 63) = 5.8, p = 0.02) on time spent in the middle chamber, with 150 mg/kg metformin-treated 129S females spending more time in the middle chamber (mean ± SD = 532 ± 187 s) than 150 mg/kg males (292 ± 269 s, p = 0.01). Supplementary Figures S1–S3 show dose-specific chamber or sniffing time preferences for each sex and strain.

### Social novelty preference

#### BTBR mice

For BTBR mice, there were no significant interactions or effects of sex or metformin dose on time spent in each chamber (Figure 2A) or on social sniffing (Figure 2B) during the novelty preference tests. However, since the sniffing time interaction (F (2, 69) = 2, p = 0.11) approached significance, an exploratory *post hoc* analysis suggested a trend toward greater novelty preference in control males than in males treated with 150 mg/kg metformin (p = 0.01) and control females (p = 0.05). No differences were evident for BTBR time in the middle chamber (mean ± SD = 78 ± 66 s). For chamber entries, there was a significant effect of dose (F (2, 69) = 4.6, p = 0.01) due to BTBR female controls making more chamber entries than females on 75 (p = 0.01) or 150 mg/kg (p = 0.03) metformin, or control males (p < 0.01) during social novelty preference tests (Figure 2C). There were no treatment or sex differences in the number of fecal boli in the arena center (mean ± SD = 7 ± 3) after testing.

**FIGURE 2:**
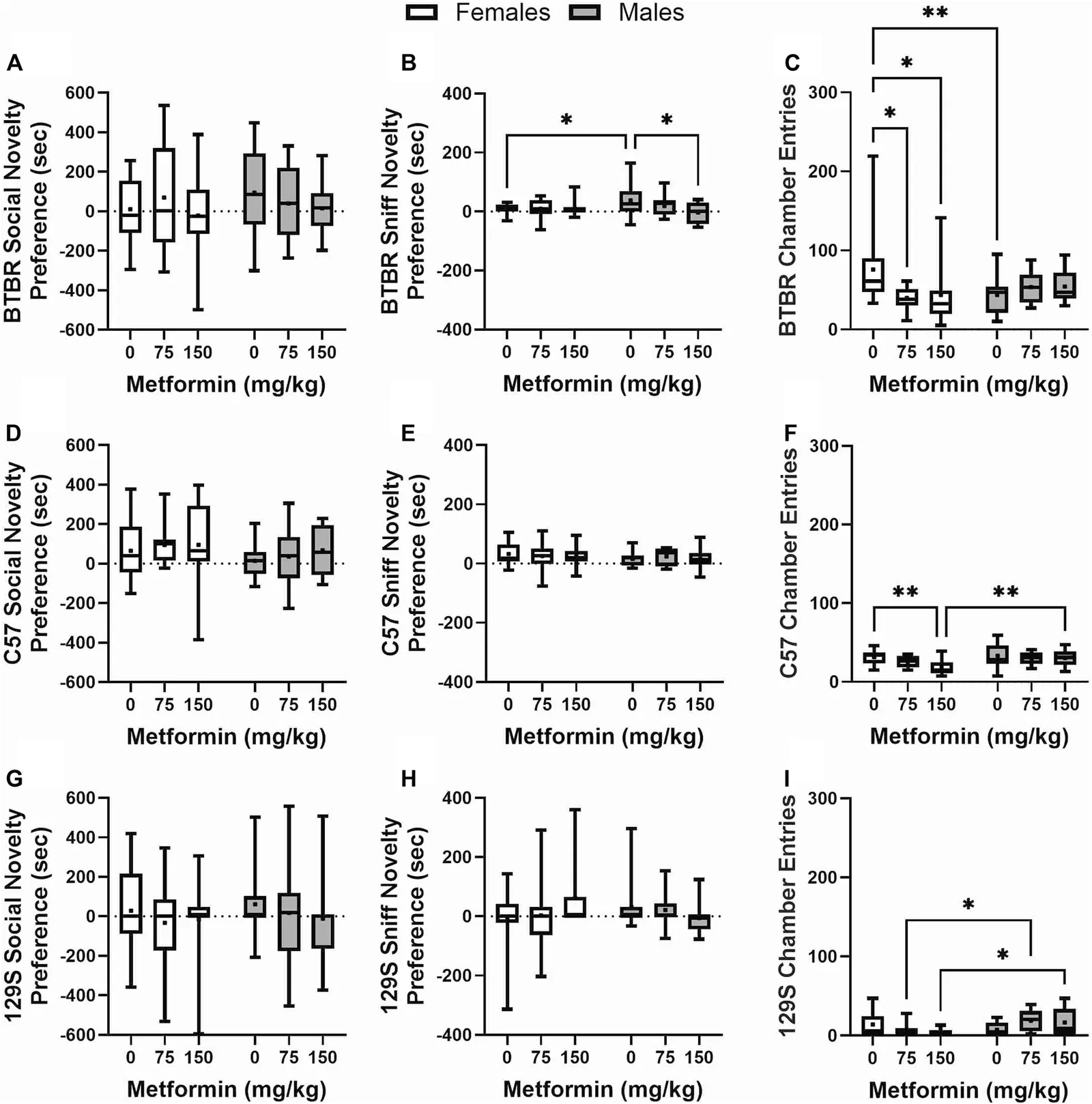
Social novelty preferences, based on differences in the time spent in chambers with or sniffing new stranger mice versus “old“ stranger mice that were previously encountered in interaction preference tests, and chamber entries of BTBR, C57 or 129S mice 65-75 min after acute metformin treatments. BTBR mice did not exhibit responses to metformin based on the differences in **(A)** time spent in chambers with or **(B)** time spent sniffing new versus old stranger mice, but in BTBR female controls made **(C)** more chamber entries than females on 75 or 150 mg/kg metformin (*p < 0.05) and BTBR male controls (**p < 0.01). C57 mice also did not show any effects of metformin treatment based on the differences in **(D)** time spent in chambers with or **(E)** time spent sniffing new versus old strangers. However, C57 females on 150 mg/kg metformin exhibited **(F)** decreased exploratory locomotion, since they made fewer chamber entries than controls or males on 150 mg/kg metformin (**p < 0.01). Metformin treatment effects in 129S mice were absent for **(G)** time spent in chambers or **(H)** sniffing, but **(I)** males entered chambers more than females on metformin (*P < 0.05). Data format and sample sizes are the same as Figure 1.

#### C57 mice

For C57 mice, there were no significant interactions, sex, or metformin dose effects based on time spent in each chamber (Figure 2D) or social sniffing (Figure 2E) during novelty preference tests. No differences were present for C57 time in the middle chamber (mean ± SD = 129 ± 71 s). For C57 chamber entries (Figure 2F), the main effects were significant for metformin dose (F (2, 66) = 3.7, p = 0.03) and sex (F (1, 66) = 6.6, p = 0.01), since 150 mg/kg treated females made fewer chamber entries than control females (p < 0.01) and 150 mg/kg metformin-treated males (p < 0.01). After both rounds of social preference tests, the number of fecal boli in the arena center (mean ± SD = 4 ± 2) did not differ among the treatment groups or sexes.

#### 129S mice

For 129S mice, no differences were found in time spent in the chambers (Figure 2G) or in time spent sniffing (Figure 2H) in social novelty preference tests. No significant group differences were detected in the time 129S mice spent in the middle chamber (mean ± SD = 331 ± 258 s). For chamber entries by 129S mice, there was a sex × dose interaction (F (2, 60) = 4.2, p = 0.02), and the effect of sex (F (1, 60) = 3.4, p = 0.07) approached significance. While there was no difference between males and females in the control group, more chamber entries were made by males than females on 75 mg/kg (p = 0.03) and 150 mg/kg (p = 0.03) metformin during social novelty preference tests (Figure 2I). Fecal boli in the arena center (mean ± SD = 4 ± 2) did not differ among treatment groups after both rounds of social preference tests for 129S mice. Supplementary Figures S4–S6 show dose-specific chamber or sniffing time preferences for novel versus original strangers, as measured by time in chambers and sniffing, for each sex and strain.

### Marble burying

For BTBR mice, no differences among metformin dose or sex were found for marbles buried after 30 min (Figure 3A). However, C57 females on 150 mg/kg metformin buried fewer marbles than control and 75 mg/kg females (F (2, 66) = 7.5, p < 0.01, Figure 3B). 129S control females buried fewer marbles than control males (F (1, 53) = 17.42, p = 0.0001), and females at each dose of metformin buried fewer marbles than males at the same doses (p < 0.05, Figure 3C).

**FIGURE 3:**
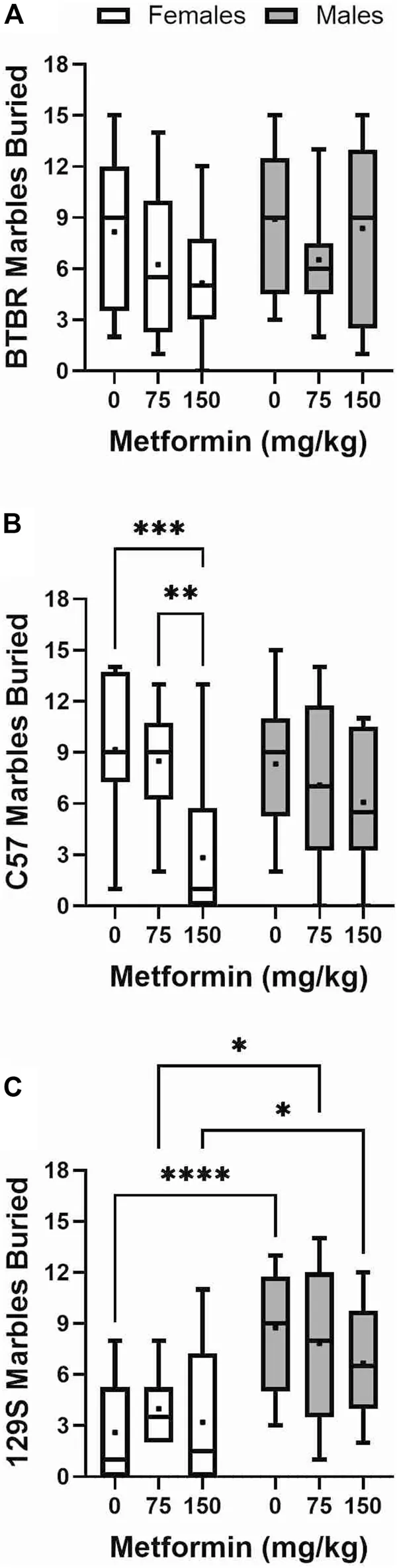
Marbles buried within 70–100 min after metformin or vehicle injections in BTBR, C57, and 129S mice. **(A)** No metformin effects were evident in BTBR mice for marbles buried withing 30 min **(B)** C57 females on 150 mg/kg metformin buried fewer marbles than controls (***p < 0.001) or 75 mg/kg (*p < 0.01) females. **(C)** 129S females buried fewer marbles than males on the vehicle control (****p < 0.0001) and on 75 mg or 150 mg/kg metformin (*p < 0.05). The data format and sample sizes are the same as in Figure 1.

### Serum steroid hormones

Progesterone levels in BTBR mice did not differ by metformin dose or sex (Figure 4A). However, in C57 mice, there was a significant interaction (F (2, 45) = 3.2, p = 0.05), wherein females on 75 mg/kg metformin had significantly higher progesterone levels than males (p < 0.01) on 75 mg/kg metformin. Females on 75 mg/kg metformin also had significantly higher progesterone levels (p = 0.03) than females on 150 mg/kg metformin (Figure 4B).

**FIGURE 4:**
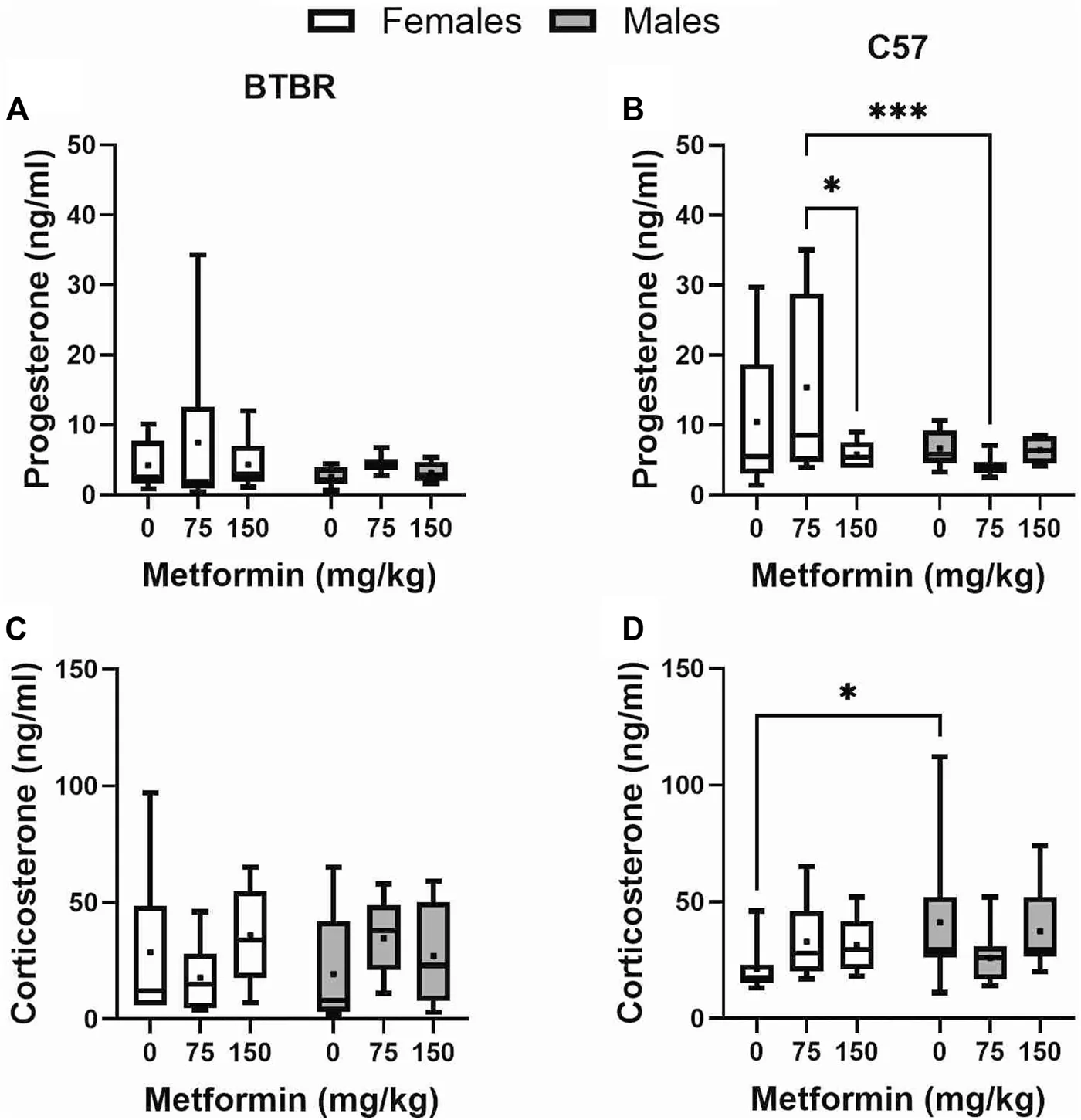
Steroid hormones in BTBR or C57 serum collected 180 min after metformin or vehicle injections. Progesterone levels in **(A)** BTBR mice were not altered by metformin treatment, but in **(B)** C57 females on 75 mg/kg metformin, progesterone levels were elevated relative to males on 75 mg/kg metformin (***p < 0.001) and females on 150 mg/kg metformin. Corticosterone levels in **(C)** BTBR male and female mice were not influenced by metformin treatment, but **(D)** control C57 females had lower serum corticosterone levels than control males (*p < 0.05). The sample sizes were 6–8 for BTBR females, 4-12 for BTBR males, 6-9 for C57 females, and 8-10 for C57 males. The data format is the same as in Figure 1.

BTBR corticosterone levels did not differ with metformin treatment or sex (Figure 4C). In C57 mice, there was a modest, non-significant trend toward an interaction (F (2, 45) = 2.6, p = 0.09) for corticosterone levels. An exploratory *post hoc* analysis revealed that this trend may have been driven by the potential (P = 0.02) for higher corticosterone levels in C57 males than in females (Figure 4D).

## Discussion

Overall, it appears that the acute treatment of 150 mg/kg metformin enhanced the social interaction preferences of BTBR male mice, a mouse model of behaviors relevant to autism. While previous research has focused on social improvements after chronic treatment, this study found that this beneficial effect was present after acute treatment with 150 mg/kg metformin [2, 26–28]. However, this effect only occurred in male BTBR mice, with females showing no improvements after metformin treatment. BTBR males treated with150 mg/kg metformin spent more time interacting with stranger mice and sniffed them more than BTBR females (at the same dose) and BTBR control males. The increase in social interaction preferences in BTBR male mice is consistent with previous findings [2, 26, 28]. The C57 positive control strain and 129S negative control strain did not show any increases in social interaction or changes in novelty after acute metformin treatment.

Previous studies have solely focused on male mice and have not tested the acute or chronic effects of metformin in females [2, 27, 28]. However, based on the results of this study, C57 and 129S strains appear to respond differently to metformin treatments. While social interaction improved in BTBR male mice, female C57 mice appeared to have exploratory or locomotor deficits after acute 150 mg/kg metformin treatment. During social interaction preference tests, this metformin treatment reduced the number of chamber entries by C57 females. Reductions in social novelty preference in sniffing behavior were observed in 150 mg/kg metformin-treated BTBR males that sniffed the original stimulus mouse more than the controls. However, there was no effect of metformin on novelty preference in C57 or 129S mice. These results lend support to the hypothesis that the social behaviors of males with ASD may benefit more from metformin treatment than those of females with ASD. The locomotor activity of 129S mice was sparse, and their preference times were variable.

Metformin did not reduce restrictive-repetitive marble-burying behavior in BTBR or 129S mice. This is consistent with the results of [28], who also did not find metformin to be effective at reducing marble burying in BTBR male mice after chronic treatment. However, these results differ from those reported by [2, 26], who did find a reduction in marble burying in BTBR males after chronic metformin treatment. In C57 mice, metformin treatment was found to decrease marble burying in female mice at 150 mg/kg metformin, whereas no change was observed in marble burying in male mice. This sex-specific effect is consistent with [2]. These results provide further evidence of sex differences in behavioral responses to metformin treatment. Marble burying by 129S mice was greater in males than in females, and metformin treatment did not affect this behavior.

Progesterone and corticosterone levels were measured in the serum since both hormones are products of the hypothalamic-pituitary-adrenal axis and can respond acutely to stress and drug treatments [34]. Progesterone levels were more variable in female BTBR and C57 mice than in males. Since the estrous stage was not controlled for in this study, the underlying cause of the significant elevation in progesterone levels in 75 mg/kg metformin-treated C57 females remains unclear. The progesterone levels recorded in this study are consistent with prior measurements of this hormone in C57 females [35]. Corticosterone levels in both strains were not influenced by acute metformin treatment, although they were higher in male C57 versus female controls. In a prior study, 150 mg/kg of oral metformin treatment for 30 days was found reduced corticosterone levels in a different mouse strain [36]. Given this, a longer exposure to metformin may be required to reduce corticosterone levels [22]. This finding also indicates that the social preference-enhancing properties of metformin are not due to changes in circulating corticosterone.

Several additional proposed mechanisms of metformin action include changes in the gut microbiome via the gut-brain axis, reduction of inflammation in the brain and body, alterations in peroxisome proliferator-activated receptors (PPARs) that modulate metabolic activity and insulin signaling in the body, and other means of regulating steroid hormones that are typically elevated in individuals with ASD [1, 6, 26–28]. However, these mechanisms will likely require longer-term treatment with metformin. Other potential mechanisms by which metformin could improve social interaction preferences in BTBR mice include AMPK or ERK activation, mTOR signaling, synaptic markers, inflammatory mediators, and immediate early gene expression [19, 37].

There are several important limitations to acknowledge in this study. First, the small sample size of mice used for behavioral testing may explain why several effects approached but did not reach significance. For the 3-chamber test, in particular, the measurements have more variance, which often requires a larger sample size. While positive effects were found in male mice, not for females, in this study, exploring metformin treatment with a larger sample of female mice would provide more clarity on its effects. Additionally, in the 3-chamber test, the only measure of locomotor behavior was the number of chamber entries made, with marble burying as the only subsequent test to determine whether metformin negatively impacts motor movement or control. Future studies should take this into consideration to determine the wide range of behavioral effects metformin could have. Furthermore, the estrous cycle of the female mice was not monitored before social behavior was measured. However, since metformin has an impact on steroid hormones, progesterone levels were measured using an ELISA kit, and amounts were higher and more variable in females. In the future, a combination of monitoring the estrous cycle and measuring steroid hormones would provide a more comprehensive view of how social behavior is impacted by female hormone cycles and metformin treatment. Finally, this study only used an acute dose of metformin, which was administered to the mice shortly before testing. It takes time for metformin to exert its full effect on the body, with saturated levels occurring after 5 half-life times [34]. Since the metformin would have only been administered once, it is likely that it would take more time and a chronic dose for it to reach its full effect. This study was designed to measure the acute effect and determine at what dose metformin could start to induce improvement, but the authors acknowledge that chronic treatment effects may differ.

Metformin has been shown to effectively reduce anxiety, improve social behavior, and, in some cases, reduce restrictive-repetitive behaviors in rodents [1, 2, 26–28]. It has also been shown to boost cognitive function in rodents and *Drosophila* [38, 39]. However, not all studies have reported positive effects of metformin. For example [40], reported impairments in cognitive function in children after metformin treatment. The results of their study highlighted the importance of considering sex and strain when evaluating the effectiveness of metformin treatment, with caution advised on its use in females, as female mice developed social deficits. However, this study supported positive effects in male BTBR mice, consistent with other studies advocating for the therapeutic use of metformin to improve social behavior [1, 2, 26–28].

## Conclusion

Acute metformin treatment improved the social behavior of male, but not female, BTBR mice. The sociability of C57 female mice did not improve after acute metformin treatment, as they did not spend any more time interacting with the stranger mouse than with the novel object. 129S mice were unaffected by the metformin treatment and did not show any changes in social behavior. For the reduction of restrictive-repetitive behavior, only C57 females buried fewer marbles after acute metformin treatment, while BTBR, 129S, and male C57 mice were unaffected.

## Data Availability

The raw data supporting the conclusions of this article will be made available by the authors, without undue reservation.
