# Supplementary material for: Acute metformin treatment increased social sniffing in male but not female BTBR mice
Source: Adv Drug Alcohol Res. 2026 Oct 12;6:16883. doi: 10.3389/adar.2026.16883 (PMC13696365; doi:10.3389/adar.2026.16883)
Supplement: Supplementary file 1 [file Table1.docx]

**SUPPLEMENTARY MATERIAL**

**Acute Metformin Treatment Increased Social Sniffing in Male, But Not Female, BTBR Mice**

Susan M Greene^1,2^, Gloria-Andrea Alcala^1,3^, Blanka Bordas^1,4^, Vy Vu^1^, Yatzil Sanchez^1^, Angela Ontanon^1,2^, Georgianna G Gould^1*^

Data shown in the main manuscript compares and shows in graphs the means of the differences in time spent in chambers with or sniffing stranger mice – novel objects for the social interaction preference tests, or differences in time spent in chambers with or sniffing novel stranger mice – original or “old” stranger mice for the social novelty tests. However, they do not reveal which mouse treatment groups had significant preferences for either the stranger mouse or the novel stranger. The purpose of this supplement is to use the raw data of time in chambers and time spent sniffing to make comparisons between chamber side times to determine which groups exhibit significant preferences. Males and females are considered separately and the times in each end of the three chamber tests are joined by lines, so the choices of each individual animal are apparent. Two-way (metformin dose x chamber side) analysis of variance with Šídák's multiple comparisons test to further discern any significant differences.

Based on time in chambers female BTBR mice in all metformin treatment groups did not exhibit any preference for stranger mice versus novel objects (Fig. S1a). Based on sniffing time, control and 75 mg/kg metformin treated female BTBR mice exhibited a significant (F (1, 33) = 13, p < 0.01) preference for stranger mice versus novel objects (Fig. S1b). There was a significant interaction (F (2, 36) = 4.4, p = 0.02) such that only male BTBR mice on 150 mg/kg metformin exhibited preference for social interaction (p < 0.01) based on time in chambers (Fig. S1c). Based on sniffing time male BTBRs treated with either dose of metformin (F (2, 36) = 6.3, p < 0.01) had a significant preference for strangers (Fig. S1d).

**Fig. S1.** Social interaction chamber times and sniffing preferences of BTBR mice in interaction preference tests. Data shown are circles = females, squares = males, open = novel object, black = stranger, lines connect time choice data for individual subject mice. (a) Females did not exhibit interaction preferences based on time in chambers, however based on (b) sniffing time control and 75 mg/kg metformin treated females preferred to sniff strangers versus novel objects (*p < 0.05). Only (c) male BTBR mice on 150 mg/kg metformin exhibited a significant preference for the chamber side with the stranger mouse (***p < 0.01). Based on sniffing time BTBR males on 75 mg/kg (*p <0.05) and 150 mg/kg metformin (***p < 0.01) had preference for stranger mice (N = 12 females, 13 males).

Control female C57 preferred strangers (F (1, 33) = 6.8, p < 0.01) based on chamber time (Fig. S2a). Control and 75 mg/kg metformin treated C57 females preferred strangers (F (1, 33) = 42, p < 0.01) based on sniffing time (Fig. S2b). Control and 75 mg/kg treated C57 males preferred strangers (F (1, 33) = 25, p < 0.01), but 150 mg/kg treated did not (Fig. S2c). Based on sniffing, all C57 male groups preferred strangers (F (1, 33) = 121, p < 0.01) over novel objects (Fig. S2d).

**Fig. S2**. Social interaction chamber time and sniffing preferences of C57 mice in interaction preference tests. Data are as described in Fig. S1. (a) Female controls preferred strangers based on chamber times (*p<0.05), and (b) all females preferred sniffing strangers (***p < 0.001, **p<0.01, *p<0.05). (c) Male controls (**p < 0.01) and 75 mg/kg treated C57 preferred strangers (*p < 0.01) based on chamber time. Based on sniffing time all C57 males (***p < 0.01) preferred stranger mice (N = 12 females or males).

Female 129S mice did not exhibit any preference for stranger mice versus novel objects in (Fig. S3a) time spent in chambers or (Fig. S3b) time sniffing during social interaction tests. Male 129S mice also did not exhibit preference for strangers in (Fig. S3c) time in chambers or (Fig. S3d).

**Fig. S3.** Social interaction chamber time and sniffing preferences of 129S mice in interaction preference tests. Data are as described in Fig. S1. Females had no preference for strangers in (a) time spent in chambers or (b) sniffing strangers, N = 10. Males also had no stranger preference based on (c) chamber or (d) sniffing times, N = 12. Many 129S mice spent most of the time in the middle chambers.

In social novelty preference tests, BTBR females did not prefer new stranger based on (Fig. S4a) chamber or (Fig. S4b) sniffing times. For time in chambers, male BTBR did not prefer novel strangers (Fig. S4c), and only control males (F (1, 36) = 6.6, p=0.01) preferred sniffing new strangers (Fig. S4d).

**Fig. S4.** Social novelty chamber times and sniffing preferences of BTBR mice in interaction preference tests. Data are circles = females, squares = males, open = novel object, black = original stranger, grey = novel stranger, lines connect time choice data for individual subject mice. Female BTBR did not prefer novel strangers with (a) time in chambers, or on (b) sniffing time. Male BTBR mice did not prefer novel strangers based on time in chambers, but by measure of sniffing time BTBR male controls did (*p <0.05).

Social novelty preference tests of C57 females found a chamber time effect (F (1, 33) = 9.5, p < 0.01), but no post hoc test preferences (Fig. S5a). Control C57 females prefered (F (1, 33) = 17, p < 0.01) for novel strangers in sniffing time (Fig. S5b). C57 males had no preference for novel strangers based on chamber time (Fig. S5c), but males on 75 mg/kg metformin preferred sniffing novel strangers (Fig. S5d).

**Fig. S5.** Social novelty chamber times and sniffing preferences of C57 mice in novelty preference tests. Data description same as in Fig. S4. Female C57 did not prefer novel strangers with (a) time in chambers, but controls did based on (b) sniffing time (*p < 0.05). Male C57 mice did not prefer novel strangers based on time in chambers, but by measure of sniffing time C57 males on 75 mg/kg metformin did (*p <0.05).

Social novelty preference tests of 129S female mice revealed no preferences for novel strangers based on time in chamber (Fig. S6a), or sniffing (Fig. S6b). Similarly, 129S males exhibited no preferences for social novelty based on times in chamber (Fig. S6c) or sniffing time (Fig. S6d).

**Fig. S6**. Social novelty chamber times and sniffing preferences of 129S mice in social novelty preference tests was highly variable. Data description same as in Fig. S4. Females did not prefer novel strangers with (a) time in chambers, or (b) sniffing time. Males did not prefer novel strangers based on time in chambers (c), or by measure of sniffing time (d).
